# Ambient Particulate Matter Air Pollution and Venous Thromboembolism in the Women’s Health Initiative Hormone Therapy Trials

**DOI:** 10.1289/ehp.1002256

**Published:** 2010-10-29

**Authors:** Regina A. Shih, Beth Ann Griffin, Nicholas Salkowski, Adria Jewell, Christine Eibner, Chloe E. Bird, Duanping Liao, Mary Cushman, Helene G. Margolis, Charles B. Eaton, Eric A. Whitsel

**Affiliations:** 1 RAND Corporation, Arlington, Virginia, USA; 2 Division of Biostatistics, School of Public Health, University of Minnesota, Minneapolis, Minnesota, USA; 3 RAND Corporation, Santa Monica, California, USA; 4 Department of Public Health Sciences, College of Medicine, Penn State University, Hershey, Pennsylvania, USA; 5 Department of Medicine, College of Medicine, University of Vermont, Burlington, Vermont, USA; 6 Department of Internal Medicine, School of Medicine, University of California at Davis, Sacramento, California, USA; 7 Department of Family Medicine and Community Health, Alpert Medical School, Brown University, Providence, Rhode Island, USA; 8 Departments of Epidemiology and Medicine, Gillings School of Global Public Health and School of Medicine, University of North Carolina, Chapel Hill, North Carolina, USA

**Keywords:** air pollution, deep vein thrombosis, particulate matter, pulmonary embolism, women’s health

## Abstract

**Background:**

The putative effects of postmenopausal hormone therapy on the association between particulate matter (PM) air pollution and venous thromboembolism (VTE) have not been assessed in a randomized trial of hormone therapy, despite its widespread use among postmenopausal women.

**Objective:**

In this study, we examined whether hormone therapy modifies the association of PM with VTE risk.

**Methods:**

Postmenopausal women 50–79 years of age (*n* = 26,450) who did not have a history of VTE and who were not taking anticoagulants were enrolled in the Women’s Health Initiative Hormone Therapy trials at 40 geographically diverse U.S. clinical centers. The women were randomized to treatment with estrogen versus placebo (E trial) or to estrogen plus progestin versus placebo (E + P trial). We used age-stratified Cox proportional hazard models to examine the association between time to incident, centrally adjudicated VTE, and daily mean PM concentrations spatially interpolated at geocoded addresses of the participants and averaged over 1, 7, 30, and 365 days.

**Results:**

During the follow-up period (mean, 7.7 years), 508 participants (2.0%) had VTEs at a rate of 2.6 events per 1,000 person-years. Unadjusted and covariate-adjusted VTE risk was not associated with concentrations of PM < 2.5 μm (PM_2.5_) or < 10 μm (PM_10_)] in aerodynamic diameter and PM × active treatment interactions were not statistically significant (*p* > 0.05) regardless of PM averaging period, either before or after combining data from both trials [e.g., combined trial-adjusted hazard ratios (95% confidence intervals) per 10 μg/m^3^ increase in annual mean PM_2.5_ and PM_10_, were 0.93 (0.54–1.60) and 1.05 (0.72–1.53), respectively]. Findings were insensitive to alternative exposure metrics, outcome definitions, time scales, analytic methods, and censoring dates.

**Conclusions:**

In contrast to prior research, our findings provide little evidence of an association between short-term or long-term PM exposure and VTE, or clinically important modification by randomized exposure to exogenous estrogens among postmenopausal women.

Under the Clean Air Act (1990), the U.S. Environmental Protection Agency (EPA) is legislatively required to establish and enforce National Ambient Air Quality Standards (NAAQS) that have a margin of safety requisite to protect the health of the public (U.S. EPA 2009a). In establishing the margin of safety for ambient particulate matter (PM) < 2.5 μm (PM_2.5_) and < 10 μm (PM_10_) in aerodynamic diameter, the U.S. EPA and the Clean Air Scientific Advisory Committee recently examined both innate (e.g., genetic or developmental) and acquired (e.g., disease- or treatment-related) forms of susceptibility to its adverse health effects, including increased risk of cardiovascular disease (U.S. EPA 2009b). Antihyperlipidemics and estrogens are noteworthy examples of the drug classes that were examined with treatment-related susceptibility to such effects in mind.

Several studies suggest that drugs in these classes modify the autonomic, atherosclerotic, and thrombotic effects of PM (Allen et al. 2009; Baccarelli et al. 2008; Künzli et al. 2005; Schwartz et al. 2005). In addition, in a case–control study set in northern Italy, Baccarelli et al. (2008) suggested that the association between PM and deep vein thrombosis is much weaker among women than among men, particularly among those women who were using hormone therapy. However, the existence of an interaction between PM and hormone therapy remains questionable because of the well-known potential for confounding associated with hormone therapy exposures in observational epidemiologic studies (Col and Pauker 2003; Hernán et al. 2008; Ray 2003). At the same time, the relative risk and severity of adverse PM effects experienced by both hormone therapy users and nonusers remains important from the clinical, public health policy, and regulatory perspectives (Brook 2008a, 2008b), given the decreasing but otherwise pervasive use of hormone therapy among postmenopausal U.S. women (Hersh et al. 2004; Tsai et al. 2010). More information about a potential association between PM and venous thromboembolism (VTE) in a randomized controlled trial of hormone therapy is therefore needed.

In this study, we examined the association between PM and VTE and interaction between PM and hormone therapy in a randomized, placebo-controlled trial of estrogen (E) or estrogen plus progestin (E + P) among a large geographically and ethnically diverse population of postmenopausal women living in the United States.

## Materials and Methods

### Study population

The design of the Women’s Health Initiative (WHI) Hormone Therapy trials has been described previously (Hays et al. 2003; Stefanick et al. 2003; National Institutes of Health 1998). Briefly, 27,347 postmenopausal women 50–79 years of age in areas surrounding 40 clinical centers were enrolled in one of two randomized, double-blind trials between 1993 and 1998. In the E trial, which was open only to women without a uterus, participants were randomized to either oral conjugated equine estrogen (CEE) 0.25 mg/day or placebo. In the E + P trial, women were randomized to CEE 0.625 mg/day plus oral medroxyprogesterone acetate 2.5 mg/day or placebo. Exclusion criteria differed slightly between the trials but were related to the presence of medical conditions associated with shortened survival or safety in both. Because of evidence of hormone therapy-related increases in VTE risk, women with a history of deep vein thrombosis or pulmonary embolism were no longer enrolled in either trial as of July 1997. The present study excluded 93 women who had no follow-up data, 312 women who reported a history of VTE at randomization, and 11 women who reported using anticoagulants or vitamin K. We also excluded 481 women with foreign addresses outside the United States, U.S. military, U.S. protectorate, Hawaiian, Alaskan, or missing addresses at the time of randomization or their event, all of which precluded estimation of PM exposures; a total of 26,450 women were included in the PM_10_ analyses. Analyses of PM_2.5_ excluded another 124 women who had a VTE event and 1,693 who dropped out of the trials before 1999, because PM_2.5_ data before 1999 were unavailable (see “Environmental exposures”). The recruitment, consent, and data collection processes of the study are overseen by institutional review boards at the Clinical Coordinating Center in the Fred Hutchinson Cancer Research Center and the 40 WHI clinical centers. All participants provided written informed consent.

### VTE event ascertainment

Study participants were followed up to assess clinical events every 6 months and for an annual in-clinic visit (Curb et al. 2006; Cushman et al. 2004). All hospital records associated with possible VTE events were locally reviewed by trained physician adjudicators who were masked to treatment assignment and then centrally adjudicated. A VTE event was defined as a centrally adjudicated deep vein thrombosis or pulmonary embolism that occurred between the date of randomization and 31 December 2004 (Curb et al. 2006); our primary outcome was the calendar date of the first VTE event postrandomization. Agreement between local and central adjudication was 97% (Curb et al. 2006). A diagnosis of deep vein thrombosis required documentation of a treating physician’s diagnosis and positive results of Doppler or duplex ultrasonography, venography, plethysmography, or isotope scanning. A pulmonary embolism required documentation of a physician’s diagnosis and by positive results from ventilation-perfusion lung scanning, pulmonary angiography, or computed tomography or by documented signs and symptoms suggestive of pulmonary embolism in the presence of a documented deep vein thrombosis event. Both procedure-related (defined as events that occurred within 60 days after an invasive procedure) and nonprocedure-related events were included in the definition of VTE events for this study (Curb et al. 2006; Cushman et al. 2004).

### Environmental exposures

We used data on the ambient concentrations of PM recorded at all U.S. EPA Air Quality System monitors operating in the contiguous United States and national-scale, log-normal ordinary kriging to spatially interpolate daily mean PM concentrations at each geocoded participant address during the study period (Liao et al. 2006; Whitsel et al. 2006). Women were assigned time-varying PM measures for all calendar dates on which a VTE event occurred. Monitor data were available for PM_10_ and PM_2.5_ between 1993–2004 and 1999–2004, respectively. We averaged spatially interpolated daily means over 1, 7, 30, and 365 days up to and including the event date among women with VTE and among women who remained at risk at that date. Averaging times were left-truncated for the cohort because of the lack of PM data before randomization. Thus, the 7-day, 30-day, and 365-day average concentrations associated with events that occured less than 7, 30, and 365 days after the randomization date were not calculable. We computed corresponding averaging times for daily mean temperature (degrees centigrade) using ambient temperature data recorded at all National Climatic Data Center meteorological stations within 50 km of each geocoded address of the participants.

### Participant and neighborhood characteristics

We examined the following participant characteristics as possible confounders of the association between PM and VTE: age (years); race/ethnicity (non-Hispanic black, non-Hispanic white, Hispanic, and other); education (four categories); history of coronary heart disease, peripheral arterial disease, or stroke; any cancer excluding nonmelanomatous skin cancers; femur or hip fracture; hypercholesterolemia; use of aspirin or nonsteroidal anti-inflammatory drugs (NSAIDs); current smoking; body mass index (BMI; kilograms per meter squared); and physical activity (kilocalories per week per kilogram). Self-reported education, medication use over the previous 2 weeks, medical history, and other characteristics were determined at each visit by interview and examination by trained and certified staff. Interim health events were identified via standardized medical record review and physician adjudication. Hypercholesterolemia was determined by history or use of antihyperlipidemics; peripheral arterial disease and coronary heart disease were determined by self-reported history at baseline or by medication use and incident disease during the follow-up period that was assessed through a review of medical records by trained adjudicators. We also examined a measure of census tract–level neighborhood socioeconomic status (SES) because of its inverse association with other cardiovascular outcomes in this population (Bird et al. 2009). To construct neighborhood SES values we identified 12 theoretically relevant census-tract level variables from census data, and assessed their relationships using confirmatory factor analysis (Bird et al., 2009). We created an index from the six variables that loaded most highly on the factor that most represented SES: percentage of adults older than age 25 years with less than a high school education; percentage of unemployed men; percentage of households with income below the poverty line; percentage of households receiving public assistance; percentage of households with children headed only by a female; and the median household income. We imputed missing values of baseline individual-level covariates using Imputation and Variance Estimation Software (IVEware; Raghunathan et al. 2002). All of the covariates listed above, except race/ethnicity and education, were time-varying covariates with values being updated on the calendar-time scale.

### Statistical analysis

Cox proportional hazard models analyzed time until VTE event, controlling for clustering within clinical centers. As detailed below, all models used calendar time as the time scale to minimize potential biases associated with comparing VTE risk at a given event date as a function of PM concentration. Thus, we considered the following model:


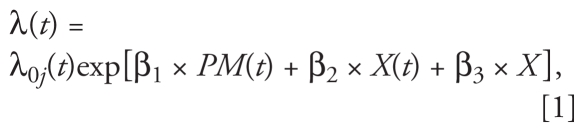


where λ_0_*_j_*(*t*) denotes the baseline hazard for the *j*th strata, *t* denotes calendar time, *PM*(*t*) denotes an individual’s PM value at time *t*, and *X*(*t*) and *X* denote the vectors containing the time-varying and time-invariant covariates used as controls in the model, respectively. We note that this model structure assumes that the effect of PM is acute, as only the value of PM at time *t* is allowed to impact risk of VTE for an individual (Haneuse et al. 2007; Krewski et al. 2000). Separate analyses were conducted for PM_10_ and PM_2.5_.

We used unadjusted models to examine the bivariate relationship between PM and VTE. Fully adjusted models controlled for trial membership (E vs. E + P), a within-trial indicator for treatment arm (active vs. placebo), time on study (days), continuous temperature (degrees centigrade), as well as the participant and neighborhood characteristics that we defined previously. Within each model, the averaging times for PM and temperature were identical. We used SAS (version 9.1.3; SAS Institute Inc., Cary, NC) to fit all models and inspected weighted Schoenfeld residual plots to test the assumption of proportional hazards for all covariates (Grambsch and Therneau 1994). Because of nonproportionality, all models were stratified by birth cohort in 5-year increments and by trial membership. All nondemographic variables were allowed to vary over the course of follow-up except for neighborhood SES and physical activity, which were measured at baseline.

All analyses began by combining data from the E and E + P trials with an indicator for active treatment in either trial. Combined trial analyses were followed by separate analyses of the E and E + P trial data. In each analysis, we first assessed the PM main effect using separate models for the 1-day, 7-day, 30-day, and 365-day average PM_10_ and PM_2.5_ concentrations and then the interactive effect between PM and treatment arm for each PM concentration. We assessed the interaction between PM and treatment arm by examining the addition of a linear PM × hormone therapy treatment arm interaction term and by comparing stratified models (E alone, E placebo, E + P, and E + P placebo) to assess nonlinear interaction effects. PM-covariate interaction terms were also examined. Statistical significance was determined using a *p*-value of 0.05.

### Time scale selection

The survival models presented above rely on a calendar time scale instead of time on study (time since randomization). When combined with time-varying PM exposure measures, the calendar time scale allows for more robust inferences concerning the association between PM and VTE. In particular, we normalized the events to the calendar time scale and computed time-varying measures of PM by time-averaging the PM data of each woman at risk of VTE for each of the actual VTE event dates. This method minimizes confounding by season and long-term trends commonly found in PM data analyses; provides a more meaningful summary of exposures of women who never experienced a VTE event (compared with simply allowing their PM exposures at loss to follow-up to represent their PM exposures over the entire follow-up period); and more appropriately models the effects of PM on VTE as short term or acute in nature.

## Results

### Characteristics of the population

Of the 26,450 women without a baseline history of VTE who met the inclusion criteria, 508 (2.0%, or 2.6/1,000 person-years) had a VTE event: 202 (2.0%, or 2.6/1,000 person-years) in the E trial (mean follow-up, 7.5 years) and 306 (1.9%, or 2.5/1,000 person-years) in the E + P trial (mean follow-up, 7.6 years). Women who experienced a VTE event during the follow-up period tended to be older and non-Hispanic white, to have had a history of stroke or cancer, to have been taking aspirin or an NSAID, and to have been randomly assigned to estrogen, with or without progestin, at baseline compared with women who did not have a VTE event during follow-up (Table 1). In Table 2 we present the mean PM concentrations for each averaging period for the overall sample and by trial arm and VTE status.

### Associations between PM and VTE and interactions between PM and hormone therapy treatment arm

Fully adjusted models that included baseline neighborhood SES data for neighborhoods were similar to those without neighborhood SES. Neighborhood SES was not significantly associated with VTE and therefore was not included in the fully adjusted models reported herein. In the combined trial analyses, unadjusted and adjusted overall hazard ratios (HRs) for VTE per 10-μg/m^3^ increase in PM_2.5_ and in PM_10_ were close to and not significantly different from 1.0, regardless of the PM averaging period (Table 3). HRs and 95% confidence intervals (CIs) for VTE associated with other model covariates in the combined trial analyses for PM_10_ are shown in Supplemental Material, Table A (doi:10.1289/ehp.1002256). Overall HRs in the trial-stratified analyses also were no different from the null, although they were somewhat higher among women in the E + P than in the E trial, except for the 30-day PM_2.5_ average (Table 4). The interaction terms between PM and randomized hormone therapy within each trial were not statistically significant (*p* > 0.05), regardless of the PM-averaging period (Table 4). Moreover, *p*-values associated with other PM-covariate interactions exceeded 0.1 (results not shown).

### Sensitivity analyses

In addition to the primary analyses, we evaluated the sensitivity of findings to alternative exposure metrics, outcome definitions, time scales, analytic methods, and trial-specific censoring dates. For example, we dichotomized 1-day and 365-day average PM concentrations at values representing current U.S. EPA regulatory standards to evaluate potential threshold effects of exposure (U.S. EPA 1990). We also examined restricted distributed lag models (Pope and Schwartz 1996; Schwartz 2000) to evaluate PM effects as a cubic function of lagged daily concentrations within the 7-day averaging period. In addition to the primary VTE outcome, pulmonary embolism (without coincident deep vein thrombosis) and deep vein thrombosis (without coincident pulmonary embolism) were also examined to evaluate their individual associations with PM and interactions between PM and hormone therapy. In other analyses, time on study was substituted for calendar time to evaluate whether use of the calendar time scale masked the true effect of PM (Mosteller and Tukey 1977). Moreover, although in the primary analyses the censoring date was 31 December 2004, the last date for which PM data were available, we examined whether associations using this date were different from those using the end dates of the two hormone therapy trials (8 July 2002 for the E trial; 1 March 2004 for the E + P trial) by restricting the follow-up time to events that occurred before those dates for each trial and rerunning all combined and trial-stratified models. Finally, we applied a case-crossover design using conditional logistic regression to analyze the odds of VTE in case compared with bidirectionally sampled case–referent exposure periods. These models controlled for short-term time trends in PM exposure and adjusted for average ambient temperature. All findings were robust to the adoption of the alternative exposure metrics, outcome definitions, time scales, analytic methods, and trial-specific censoring dates (see “Materials and Methods”). In the adjusted analyses of the 365-day average PM_2.5_ and PM_10_ on the time on study scale, for example, the combined trial HRs were consistently null: 0.88 (95% CI, 0.52–1.47) and 1.10 (95% CI, 0.75–1.61), respectively, as were the trial-stratified HRs. In the case-crossover analyses, odds ratios (95% CIs) for VTE per 10-μg/m^3^ increase in average 1-day and 7-day PM_2.5_ were 1.04 (0.85–1.28) and 0.93 (0.68–1.27), respectively. The odds ratios for a 10-μg/m^3^ increase in 1-day and 7-day PM_10_, were 1.03 (0.92–1.14) and 0.99 (0.81–1.20), respectively.

## Discussion

In this large clinical trial population, we observed little evidence for an association of either PM_10_ or PM_2.5_ air pollution with risk of future VTE, over more than 7 years of follow-up. Results were robustly null in analyses that examined daily, weekly, monthly, and annual average PM concentrations immediately preceding VTE events, adjusting for a comprehensive set of sociodemographic, clinical, behavioral, and environmental covariates. There were no differences in findings comparing women who were randomized to placebo or hormone therapy.

Although the current study did not find any consistent evidence of a direct association between VTE risk and short-term or long-term PM_2.5_ or PM_10_ exposures in primary or sensitivity analyses, Baccarelli et al. (2008) found a strong, direct, and significant association between PM_10_ exposure and the risk of deep vein thrombosis in a case–control study conducted in the Lombardy region of Italy. This finding was internally consistent with the PM-related decreases in prothrombin time and in activated partial thromboplastin time that the study also observed among its controls. Moreover, other studies have suggested links between traffic or PM_10_ exposure, deep vein thrombosis (Baccarelli et al. 2009), pulmonary embolism (Colais et al. 2009), and VTE (Dales et al. 2010). The discrepant findings may be partly attributable to differences between study populations; for example, Baccarelli et al. (2008) included both men and women in their study. They observed lower VTE risk among women than among men, suggesting that there could be inherent or acquired differences in risk for VTE by sex that we were not able to observe in our sample of women. Women in the WHI Hormone Therapy trials were also older than those reported in Baccarelli et al. (2008), uniformly postmenopausal, mostly well educated, and likely to have had a substantial interest in their health. In addition, the association between PM_10_ and deep vein thrombosis described by Baccarelli et al. (2008) was not adjusted for history of cancer, which is a risk factor for deep vein thrombosis (Anderson and Spencer 2003; Goldhaber 1998; Lutsey et al. 2009; Severinsen et al. 2009). Finally, the chemical composition of ambient PM and its correlation with personal PM exposures may differ in northern Italy and in the United States (de Meij et al. 2009; Giugliano et al. 2005; Lonati et al. 2005).

Although PM-chemical component data are currently unavailable in the WHI, we were able to examine hormone therapy-related susceptibility to the putative effects of PM on VTE risk in the context of a randomized clinical trial of estrogen with or without progestin. We did not find a consistent clinically or statistically significant interactive effect of hormone therapy. Baccarelli et al. (2008) also examined the interactive effects of PM and exogenous estrogens on risk of deep vein thrombosis with or without pulmonary embolism; although no significant interactions were found at a Bonferroni-corrected alpha of 0.004, the strong and direct association between PM and deep vein thrombosis described by the researchers was much weaker among women than among men and among users of oral contraceptives or hormone therapy compared with women who were nonusers. These findings were counterintuitive, because the prothrombotic effects of PM (Liao et al. 2005) and estrogen (Curb et al. 2006; Cushman et al. 2004; Herrington et al. 2002; Sare et al. 2008) were expected to be synergistic (Brook 2008a). However, the case–control design of the study did not benefit from the equalization among treatment groups of measured, and most important, unmeasured confounders that randomization typically affords. Studies that report protective effects of hormone therapy against coronary heart disease in observational settings conflict with more recent randomized trials of hormone therapy (Curb et al. 2006) and with reanalyses of those observational associations using methods less prone to bias (Col and Pauker 2003; Hernán et al. 2008; Ray 2003). Thus, the putative effects of hormone therapy and its interactive effects with PM on VTE are particularly important to examine in the context of a randomized trial, as has been done here, although neither PM exposure nor its interaction with hormone therapy was randomized.

Prior findings from the WHI Observational Study are useful in placing the present findings from the WHI Hormone Therapy trials into proper perspective. Those findings included a 24% (95% CI, 9–41) increase in the risk of a cardiovascular event, including death from coronary heart disease, or cerebrovascular disease, coronary revascularization, myocardial infarction, and stroke per 10-μg/m^3^ increase in the annual average PM_2.5_ concentration (Miller et al. 2007). The strength of that effect also varied in a striking and consistent way with several anthropometric measures. The generally null main and interactive effects described here contrast sharply with those of Miller et al. (2007) and suggest that observations of the WHI populations pertaining to the atherosclerotic and arterial effects of PM_2.5_ do not necessarily apply to its thromboembolic and venous effects, as has been intimated previously (Brook 2008a, 2008b).

The strengths of this study include the examination of a geographically and demographically diverse population of U.S. women who participated in a large randomized trial of postmenopausal hormone therapy, the rigor with which VTE outcomes were ascertained, and the analysis of PM_10_ as well as PM_2.5_, the particle size most relevant to regulatory efforts in the United States. Moreover, we distinguished between the effects of short-term (1-day or 7-day), medium-term (30-day), and long-term (365-day) exposures to both PM_2.5_ and PM_10_ on both pulmonary embolism and deep vein thrombosis risk, using previously validated PM exposures measured across 1,270 counties in the United States (Liao et al. 2006).

Despite these strengths, we note several limitations. First, this study is based on postmenopausal women 50–79 years of age who lived in the contiguous United States between 1993 (or 1999 for those with PM_2.5_ measures) and 2004. Thus, inferences may not be generalizable to the U.S. population of adults including men and premenopausal women. Second, the distribution of PM concentrations in this study may not be fully representative of individual PM exposures across the United States. The temporally and spatially heterogeneous PM concentrations in this study are nonetheless associated with means, variances, and ranges comparable with those recorded by the U.S. EPA over the same time frame (U.S. EPA 2009c). Third, ambient PM concentrations estimated in this study may not adequately represent total PM exposures (Avery et al. 2010a, 2010b) as participants may have been exposed to occupational or indoor PM with physicochemical properties and toxicities that differ from those of ambient PM (Monn and Becker 1999; Wainman et al. 2000). Even so, surveys of human-activity patterns suggest that individuals spend a majority of their time at home (Klepeis et al. 2001), the location where ambient PM concentrations in this study were estimated.

## Conclusions

We consistently found little evidence of an association between VTE and PM_2.5_ or PM_10_ concentrations in this study. Although innate participant characteristics remain unaccounted for in this study—including those that may influence genetic susceptibility to PM effects on VTE (Curb et al. 2006; Cushman et al. 2004; Herrington et al. 2002; Martinelli et al. 1999; Rosendaal et al. 1995)—genome-wide association studies (GWAS) are beginning to offer new opportunities to examine gene-PM interactions. Such studies hold promise for improved understanding of genetic and environmental susceptibility to VTE.

## Figures and Tables

**Table 1:** Baseline characteristics, overall and by VTE status.

	VTE status		
Characteristic	Overall (*n* = 26,450)	No VTE (*n* = 25,942)	VTE (*n* = 508)
Age (years)	63.4 ± 7.2	63.4 ± 7.2	66.1 ± 6.6
Race/ethnicity			
Non-Hispanic white	21,609 (81.7)	21,166 (81.6)	443 (87.2)
Non-Hispanic black	2,699 (10.2)	2,646 (10.2)	53 (10.4)
Hispanic	1,498 (5.7)	1,491 (5.7)	7 (1.4)
Other	644 (2.4)	639 (2.5)	5 (1.0)
Education			
Some high school	2,130 (8.0)	2,085 (8.0)	45 (8.9)
High school graduate	5,454 (20.6)	5,348 (20.6)	106 (20.9)
Some college	10,733 (40.6)	10,531 (40.6)	202 (39.7)
≥ College degree	8,133 (30.8)	7,978 (30.8)	155 (30.5)
BMI (kg/m^2^)	29.1 ± 6.0	29.1 ± 6.0	31.2 ± 6.6
Coronary heart disease	494 (1.9)	484 (1.9)	10 (2.0)
Peripheral arterial disease	440 (1.7)	429 (1.7)	11 (2.2)
Stroke	294 (1.1)	285 (1.1)	9 (1.8)
Hypercholesterolemia	3,206 (12.1)	3,146 (12.1)	60 (11.8)
Cancer	840 (3.2)	817 (3.1)	23 (4.5)
Fracture^a^	211 (0.8)	206 (0.8)	5 (1.0)
Aspirin or NSAID use	10,150 (38.4)	9,906 (38.2)	244 (48.0)
Current smoking	2,735 (10.5)	2,693 (10.5)	50 (8.4)
Physical activity (kcal/kg/week)	10.0 ± 11.2	10.0 ± 11.2	8.7 ± 10.6
Randomization status			
E	5,104 (19.3)	4,991 (19.2)	113 (22.2)
Placebo for E arm	5,228 (19.8)	5,139 (19.8)	89 (17.5)
E + P	8,255 (31.2)	8,064 (31.1)	191 (37.6)
Placebo for E + P arm	7,863 (29.7)	7,748 (29.9)	115 (22.6)
Daily temperature (°C)	NA	NA	13.0 ± 9.5
Season			
January–March	NA	NA	177 (34.8)
April–June	NA	NA	124 (24.4)
July–September	NA	NA	150 (29.5)
October–December	NA	NA	57 (11.2)
Follow-up time (person-years)	198,048	195,898	2,150

Abbreviations: E, CEE, 0.625 mg/day; E + P, CEE, 0.625 mg/day plus medroxyprogesterone acetate, 0.25 mg/day; NA, not applicable for participants without event. Data are reported as *n* (%) or mean ± SD. Descriptive data include imputed baseline covariate data.

a Hip or femur.

**Table 2:** Overall mean ± SD PM concentrations at baseline, by trial arm and by VTE status.

		E trial				E + P trial			
		Placebo		E		Placebo		E + P	
Averaging period (days)^a^	Overall (*n* = 26,450)	VTE (*n* = 89)	No VTE (*n* = 5,139)	VTE (*n* = 113)	No VTE (*n* = 4,991)	VTE (*n* = 115)	No VTE (*n* = 7,748)	VTE (*n* = 191)	No VTE (*n* = 8,064)
PM_2.5_ (μg/m^3^)									

1	13.5 ± 7.7	13.5 ± 7.7	12.5 ± 7.2	13.5 ± 7.7	12.9 ± 7.0	13.5 ± 7.8	13.0 ± 7.3	13.5 ± 7.7	15.3 ± 9.3
7	13.5 ± 5.6	13.5 ± 5.6	12.5 ± 6.1	13.5 ± 5.6	13.7 ± 5.1	13.5 ± 5.6	13.7 ± 5.9	13.5 ± 5.6	13.6 ± 5.8
30	13.4 ± 4.4	13.4 ± 4.4	12.9 ± 4.1	13.4 ± 4.4	13.6 ± 4.1	13.4 ± 4.4	13.7 ± 4.3	13.4 ± 4.4	13.6 ± 4.8
365	13.4 ± 2.9	13.4 ± 2.9	13.1 ± 2.5	13.4 ± 2.9	13.2 ± 2.8	13.3 ± 2.8	13.7 ± 2.4	13.4 ± 2.8	13.4 ± 2.9

PM_10_ (μg/m^3^)									

1	27.8 ± 12.3	27.9 ± 12.3	28.1 ± 10.9	27.9 ± 12.2	25.6 ± 9.6	27.7 ± 12.2	26.7 ± 10.3	27.7 ± 12.3	27.5 ± 12.0
7	27.1 ± 8.0	27.2 ± 8.0	26.0 ± 7.7	27.2 ± 8.0	26.4 ± 7.3	27.0 ± 7.9	26.8 ± 8.0	27.0 ± 8.0	26.5 ± 7.6
30	26.7 ± 6.1	26.8 ± 6.2	26.4 ± 6.2	26.9 ± 6.2	25.8 ± 5.3	26.7 ± 6.1	26.6 ± 6.2	26.7 ± 6.1	26.4 ± 6.0
365	26.7 ± 3.6	26.8 ± 3.7	26.7 ± 3.7	26.8 ± 3.7	26.7 ± 3.3	26.6 ± 3.6	26.8 ± 3.4	26.6 ± 3.6	26.7 ± 3.4

a Periods (up to and ending on VTE event dates) over which averages were calculated. For those without VTE, averages are the corresponding averages over all at-risk days.

**Table 3:** HRs (95% CIs) for VTE per 10-μg/m^3^ increase in PM.^a^

Averaging period (days)	Unadjusted HR (95% CI)	Adjusted HR (95% CI)
PM_2.5_		

1	1.01 (0.88–1.16)	1.04 (0.89–1.22)
7	0.97 (0.78–1.20)	1.00 (0.78–1.28)
30	0.99 (0.76–1.29)	1.02 (0.73–1.43)
365	1.03 (0.66–1.62)	0.93 (0.54–1.60)

PM_10_		

1	0.94 (0.86–1.03)	0.98 (0.88–1.10)
7	0.88 (0.76–1.02)	0.91 (0.77–1.08)
30	0.87 (0.71–1.05)	0.93 (0.74–1.16)
365	0.92 (0.65–1.29)	1.05 (0.72–1.53)

a Models were stratified by birth cohort (in 5-year increments) and trial arm. Adjusted models include race/ethnicity, education, BMI, coronary heart disease, peripheral arterial disease, stroke, cancer, fracture, aspirin or NSAID use, hypercholesterolemia, current smoking, physical activity, hormone therapy treatment arm (active vs. placebo), treatment duration (years), and temperature (degrees centigrade).

**Table 4:** HRs (95% CIs) for VTE per 10-μg/m^3^ increase in PM, by randomization status.^a^

	E trial				E + P trial			
Averaging period (days)	Total E trial	Placebo	E	*p*-Value^b^	Total E + P trial	Placebo	E + P	*p*-Value^b^
PM_2.5_								

1	0.97 (0.79–1.18)	0.94 (0.68–1.31)	0.98 (0.74–1.31)	0.86	1.10 (0.87–1.4)	0.97 (0.67–1.41)	1.18 (0.88–1.60)	0.42
7	0.98 (0.70–1.37)	0.78 (0.40 1.53)	1.13 (0.81–1.58)	0.32	1.01 (0.75–1.36)	1.12 (0.69–1.81)	0.94 (0.63–1.41)	0.60
30	1.07 (0.66–1.75)	0.76 (0.36–1.63)	1.31 (0.81–2.13)	0.14	0.98 (0.65–1.47)	1.02 (0.50–2.1)	0.95 (0.58–1.55)	0.86
365	0.67 (0.34–1.29)	0.54 (0.21–1.38)	0.77 (0.32–1.84)	0.58	1.21 (0.57–2.56)	2.07 (0.77–5.61)	0.81 (0.31–2.07)	0.12

PM_10_								

1	0.95 (0.84–1.09)	1.06 (0.93–1.22)	0.87 (0.71–1.05)	0.06	1.01 (0.86–1.18)	0.97 (0.79–1.2)	1.02 (0.86–1.22)	0.67
7	0.90 (0.70–1.15)	0.86 (0.60–1.24)	0.93 (0.68–1.26)	0.74	0.92 (0.75–1.13)	0.95 (0.70–1.28)	0.90 (0.72–1.13)	0.78
30	0.89 (0.64–1.24)	1.02 (0.65–1.59)	0.82 (0.55–1.21)	0.41	0.96 (0.72–1.29)	1.00 (0.62–1.61)	0.94 (0.70–1.26)	0.79
365	0.83 (0.50–1.38)	1.04 (0.39–2.75)	0.71 (0.41–1.24)	0.52	1.23 (0.78–1.93)	1.29 (0.65–2.53)	1.20 (0.75–1.91)	0.83

a Models were stratified by birth cohort and trial arm and adjusted for race/ethnicity, education, BMI, coronary heart disease, peripheral arterial disease, stroke, cancer, fracture, aspirin or NSAID use, hypercholesterolemia, current smoking, physical activity, treatment duration (years), and temperature (degrees centigrade).

b *p*-Value for the test of the PM × treatment interaction.

## Appendix 1. Additional Acknowledgments

The authors had full access to all of the data used in the study and take responsibility for the integrity of the data and the accuracy of the data analysis. The authors published their preliminary findings as an abstract at the American Heart Association 2009 Annual Meeting in Orlando, FL. They acknowledge the contributions of the following WHI investigators:

**Program Office** (National Heart, Lung, and Blood Institute, Bethesda, MD) E. Nabel, J. Rossouw, S. Ludlam, L. Pottern, J. McGowan, L. Ford, and N. Geller.

**Clinical Coordinating Center** (Fred Hutchinson Cancer Research Center, Seattle, WA) R. Prentice, G. Anderson, A. LaCroix, C.L. Kooperberg, R.E. Patterson, A. McTiernan; (Wake Forest University School of Medicine, Winston-Salem, NC) S. Shumaker; (Medical Research Labs, Highland Heights, KY) E. Stein; (University of California at San Francisco, San Francisco, CA) S. Cummings.

**Clinical Centers** (Albert Einstein College of Medicine, Bronx, NY) S. Wassertheil-Smoller; (Baylor College of Medicine, Houston, TX) A. Rajkovic; (Brigham and Women’s Hospital, Harvard Medical School, Boston, MA) J. Manson; (Brown University, Providence, RI) A.R. Assaf; (Emory University, Atlanta, GA) L. Phillips; (Fred Hutchinson Cancer Research Center, Seattle, WA) S. Beresford; (George Washington University Medical Center, Washington, DC) J. Hsia; (Harbor-UCLA Research and Education Institute, Torrance, CA) R. Chlebowski; (Kaiser Permanente Center for Health Research, Portland, OR) E. Whitlock; (Kaiser Permanente Division of Research, Oakland, CA) B. Caan; (Medical College of Wisconsin, Milwaukee, WI) J.M. Kotchen; (MedStar Research Institute/Howard University, Washington, DC) B.V. Howard; (Northwestern University, Chicago/Evanston, IL) L. Van Horn; (Rush Medical Center, Chicago, IL) H. Black; (Stanford Prevention Research Center, Stanford, CA) M.L. Stefanick; (State University of New York at Stony Brook, Stony Brook, NY) D. Lane; (The Ohio State University, Columbus, OH) R. Jackson; (University of Alabama at Birmingham, Birmingham, AL) C.E. Lewis; (University of Arizona, Tucson/Phoenix, AZ) T. Bassford; (University at Buffalo, Buffalo, NY) J. Wactawski-Wende; (University of California at Davis, Sacramento, CA) J. Robbins; (University of California at Irvine, CA) F.A. Hubbell; (University of California at Los Angeles, Los Angeles, CA) L. Nathan; (University of California at San Diego, LaJolla/Chula Vista, CA) R.D. Langer; (University of Cincinnati, Cincinnati, OH) M. Gass; (University of Florida, Gainesville/Jacksonville, FL) M. Limacher; (University of Hawaii, Honolulu, HI) D. Curb; (University of Iowa, Iowa City/Davenport, IA) R. Wallace; (University of Massachusetts/Fallon Clinic, Worcester, MA) J. Ockene; (University of Medicine and Dentistry of New Jersey, Newark, NJ) N. Lasser; (University of Miami, Miami, FL) M.J. O’Sullivan; (University of Minnesota, Minneapolis, MN) K. Margolis; (University of Nevada, Reno, NV) R. Brunner; (University of North Carolina, Chapel Hill, NC) G. Heiss; (University of Pittsburgh, Pittsburgh, PA) L. Kuller; (University of Tennessee, Memphis, TN) K.C. Johnson; (University of Texas Health Science Center, San Antonio, TX) R. Brzyski; (University of Wisconsin, Madison, WI) G.E. Sarto; (Wake Forest University School of Medicine, Winston-Salem, NC) M. Vitolins; (Wayne State University School of Medicine/Hutzel Hospital, Detroit, MI) S. Hendrix.
